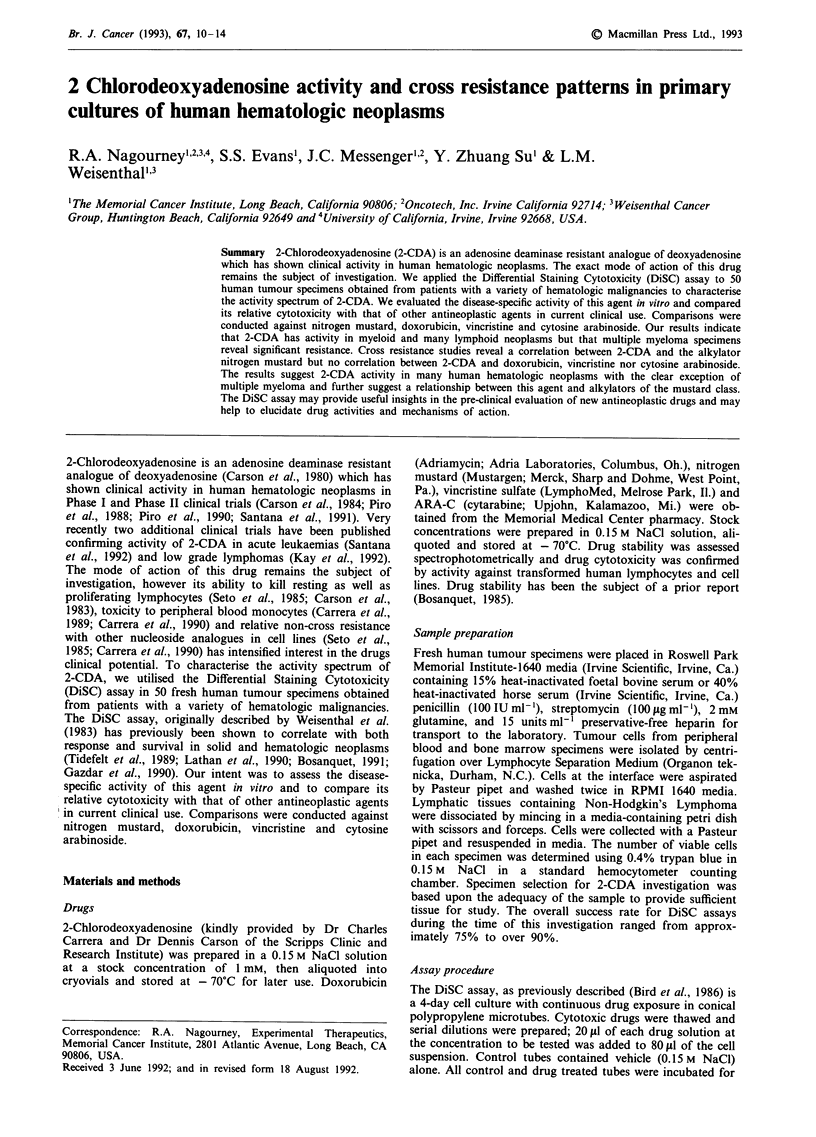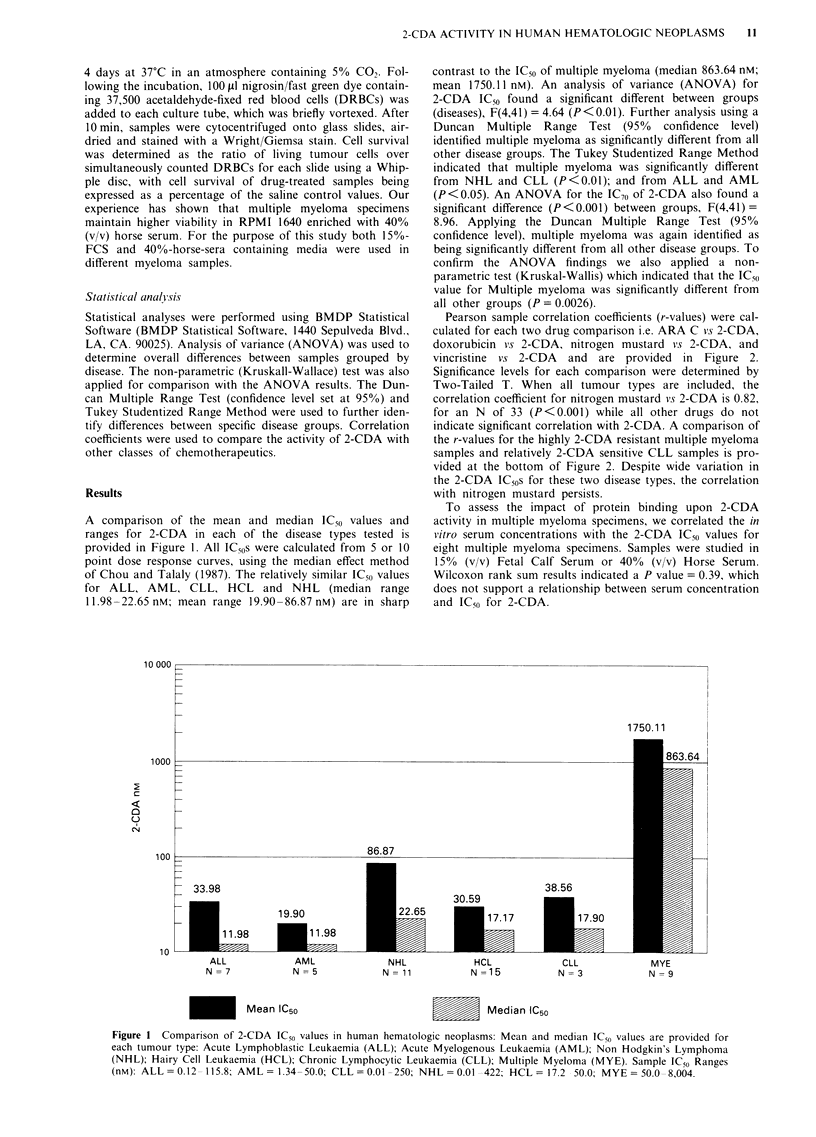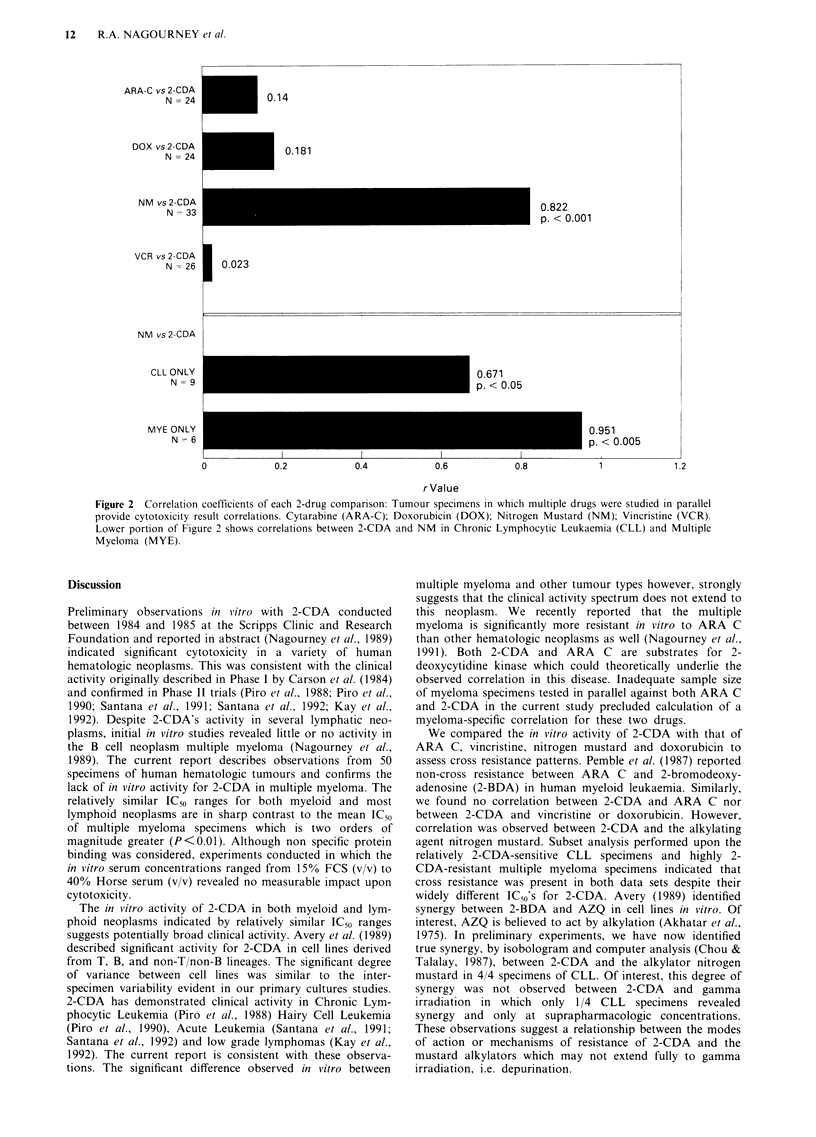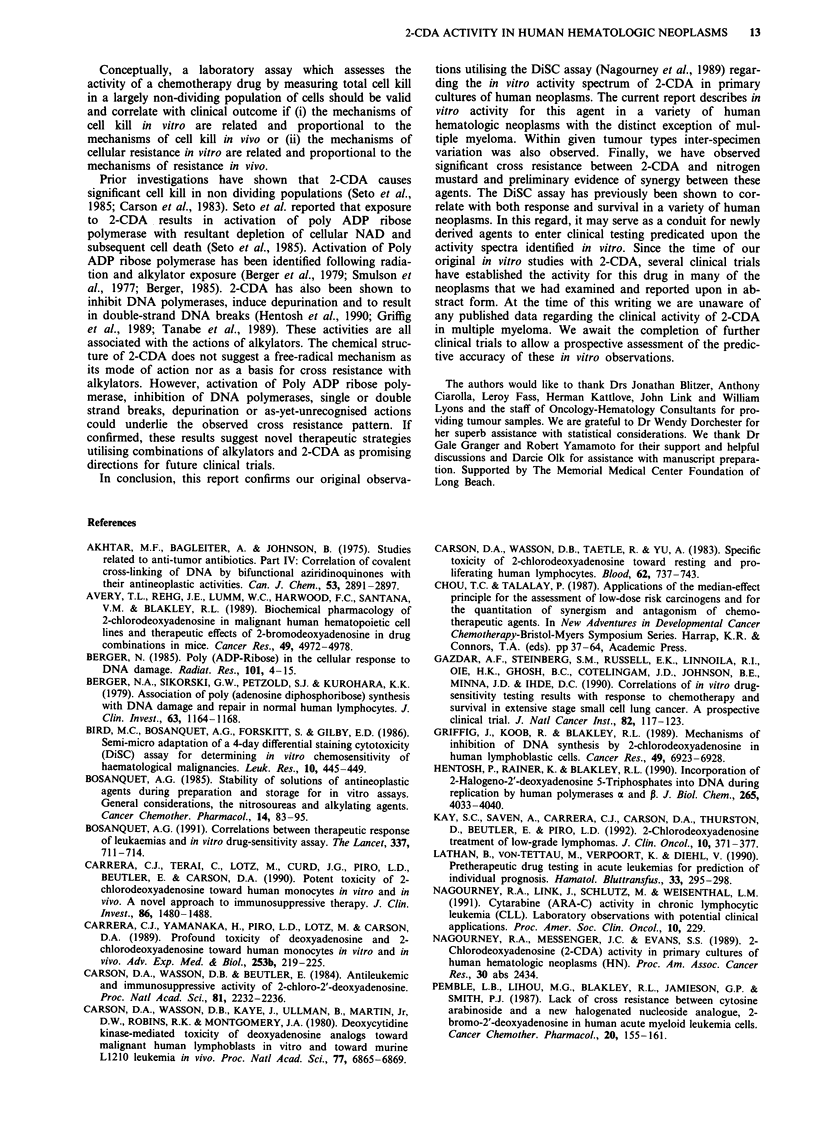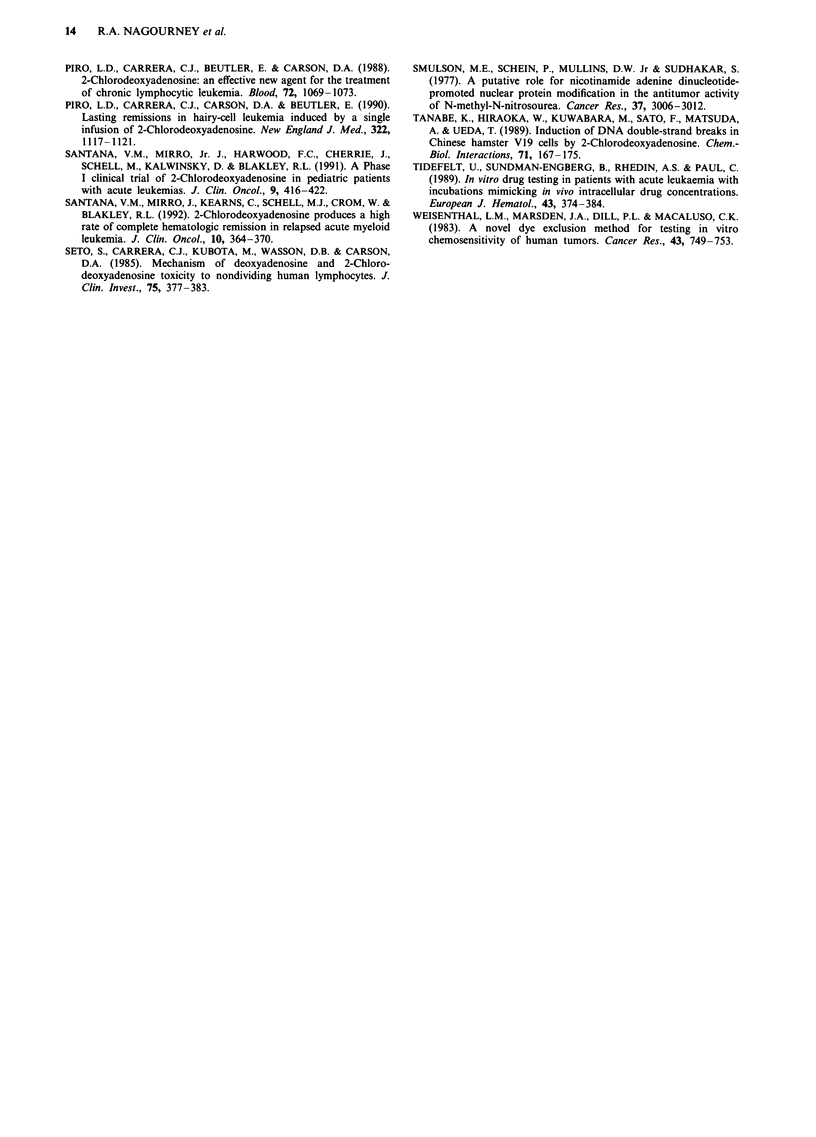# 2 chlorodeoxyadenosine activity and cross resistance patterns in primary cultures of human hematologic neoplasms.

**DOI:** 10.1038/bjc.1993.3

**Published:** 1993-01

**Authors:** R. A. Nagourney, S. S. Evans, J. C. Messenger, Y. Z. Su, L. M. Weisenthal

**Affiliations:** Memorial Cancer Institute, Long Beach, California 90806.

## Abstract

2-Chlorodeoxyadenosine (2-CDA) is an adenosine deaminase resistant analogue of deoxyadenosine which has shown clinical activity in human hematologic neoplasms. The exact mode of action of this drug remains the subject of investigation. We applied the Differential Staining Cytotoxicity (DiSC) assay to 50 human tumour specimens obtained from patients with a variety of hematologic malignancies to characterise the activity spectrum of 2-CDA. We evaluated the disease-specific activity of this agent in vitro and compared its relative cytotoxicity with that of other antineoplastic agents in current clinical use. Comparisons were conducted against nitrogen mustard, doxorubicin, vincristine and cytosine arabinoside. Our results indicate that 2-CDA has activity in myeloid and many lymphoid neoplasms but that multiple myeloma specimens reveal significant resistance. Cross resistance studies reveal a correlation between 2-CDA and the alkylator nitrogen mustard but no correlation between 2-CDA and doxorubicin, vincristine nor cytosine arabinoside. The results suggest 2-CDA activity in many human hematologic neoplasms with the clear exception of multiple myeloma and further suggest a relationship between this agent and alkylators of the mustard class. The DiSC assay may provide useful insights in the pre-clinical evaluation of new antineoplastic drugs and may help to elucidate drug activities and mechanisms of action.


Br. J. Cancer (1993), 67, 10-14 © Macmillan Press Ltd., 1993

### 2 Chlorodeoxyadenosine activity and cross resistance patterns in primary cultures of human hematologic neoplasms

R.A. Nagourney[1,2,3,4], S.S. Evans[1], J.C. Messenger[1,2], Y. Zhuang Su[1] & L.M. Weisenthal[1,3]

[1]*The Memorial Cancer Institute, Long Beach, California 90806;* [2]*Oncotech, Inc. Irvine California 92714;* [3]*Weisenthal Cancer Group, Huntington Beach, California 92649 and* [4]*University of California, Irvine, Irvine 92668, USA.*

**Summary** 2-Chlorodeoxyadenosine (2-CDA) is an adenosine deaminase resistant analogue of deoxyadenosine which has shown clinical activity in human hematologic neoplasms. The exact mode of action of this drug remains the subject of investigation. We applied the Differential Staining Cytotoxicity (DiSC) assay to 50 human tumour specimens obtained from patients with a variety of hematologic malignancies to characterise the activity spectrum of 2-CDA. We evaluated the disease-specific activity of this agent *in vitro* and compared its relative cytotoxicity with that of other antineoplastic agents in current clinical use. Comparisons were conducted against nitrogen mustard, doxorubicin, vincristine and cytosine arabinoside. Our results indicate that 2-CDA has activity in myeloid and many lymphoid neoplasms but that multiple myeloma specimens reveal significant resistance. Cross resistance studies reveal a correlation between 2-CDA and the alkylator nitrogen mustard but no correlation between 2-CDA and doxorubicin, vincristine nor cytosine arabinoside. The results suggest 2-CDA activity in many human hematologic neoplasms with the clear exception of multiple myeloma and further suggest a relationship between this agent and alkylators of the mustard class. The DiSC assay may provide useful insights in the pre-clinical evaluation of new antineoplastic drugs and may help to elucidate drug activities and mechanisms of action.

2-Chlorodeoxyadenosine is an adenosine deaminase resistant analogue of deoxyadenosine (Carson *et al.*, 1980) which has shown clinical activity in human hematologic neoplasms in Phase I and Phase II clinical trials (Carson *et al.*, 1984; Piro *et al.*, 1988; Piro *et al.*, 1990; Santana *et al.*, 1991). Very recently two additional clinical trials have been published confirming activity of 2-CDA in acute leukaemias (Santana *et al.*, 1992) and low grade lymphomas (Kay *et al.*, 1992). The mode of action of this drug remains the subject of investigation, however its ability to kill resting as well as proliferating lymphocytes (Seto *et al.*, 1985; Carson *et al.*, 1983), toxicity to peripheral blood monocytes (Carrera *et al.*, 1989; Carrera *et al.*, 1990) and relative non-cross resistance with other nucleoside analogues in cell lines (Seto *et al.*, 1985; Carrera *et al.*, 1990) has intensified interest in the drugs clinical potential. To characterise the activity spectrum of 2-CDA, we utilised the Differential Staining Cytotoxicity (DiSC) assay in 50 fresh human tumour specimens obtained from patients with a variety of hematologic malignancies. The DiSC assay, originally described by Weisenthal *et al.* (1983) has previously been shown to correlate with both response and survival in solid and hematologic neoplasms (Tidefelt *et al.*, 1989; Lathan *et al.*, 1990; Bosanquet, 1991; Gazdar *et al.*, 1990). Our intent was to assess the disease-specific activity of this agent *in vitro* and to compare its relative cytotoxicity with that of other antineoplastic agents in current clinical use. Comparisons were conducted against nitrogen mustard, doxorubicin, vincristine and cytosine arabinoside.

### Materials and methods

### *Drugs*

2-Chlorodeoxyadenosine (kindly provided by Dr Charles Carrera and Dr Dennis Carson of the Scripps Clinic and Research Institute) was prepared in a 0.15 M NaCl solution at a stock concentration of 1 mM, then aliquoted into cryovials and stored at − 70°C for later use. Doxorubicin (Adriamycin; Adria Laboratories, Columbus, Oh.), nitrogen mustard (Mustargen; Merck, Sharp and Dohme, West Point, Pa.), vincristine sulfate (LymphoMed, Melrose Park, Il.) and ARA-C (cytarabine; Upjohn, Kalamazoo, Mi.) were obtained from the Memorial Medical Center pharmacy. Stock concentrations were prepared in 0.15 M NaCl solution, aliquoted and stored at − 70°C. Drug stability was assessed spectrophotometrically and drug cytotoxicity was confirmed by activity against transformed human lymphocytes and cell lines. Drug stability has been the subject of a prior report (Bosanquet, 1985).

### *Sample preparation*

Fresh human tumour specimens were placed in Roswell Park Memorial Institute-1640 media (Irvine Scientific, Irvine, Ca.) containing 15% heat-inactivated foetal bovine serum or 40% heat-inactivated horse serum (Irvine Scientific, Irvine, Ca.) penicillin (100 IU $ml^{-1}$), streptomycin (100 $\mu g$ $ml^{-1}$), 2 mM glutamine, and 15 units $ml^{-1}$ preservative-free heparin for transport to the laboratory. Tumour cells from peripheral blood and bone marrow specimens were isolated by centrifugation over Lymphocyte Separation Medium (Organon teknicka, Durham, N.C.). Cells at the interface were aspirated by Pasteur pipet and washed twice in RPMI 1640 media. Lymphatic tissues containing Non-Hodgkin's Lymphoma were dissociated by mincing in a media-containing petri dish with scissors and forceps. Cells were collected with a Pasteur pipet and resuspended in media. The number of viable cells in each specimen was determined using 0.4% trypan blue in 0.15 M NaCl in a standard hemocytometer counting chamber. Specimen selection for 2-CDA investigation was based upon the adequacy of the sample to provide sufficient tissue for study. The overall success rate for DiSC assays during the time of this investigation ranged from approximately 75% to over 90%.

### *Assay procedure*

The DiSC assay, as previously described (Bird *et al.*, 1986) is a 4-day cell culture with continuous drug exposure in conical polypropylene microtubes. Cytotoxic drugs were thawed and serial dilutions were prepared; 20 $\mu l$ of each drug solution at the concentration to be tested was added to 80 $\mu l$ of the cell suspension. Control tubes contained vehicle (0.15 M NaCl) alone. All control and drug treated tubes were incubated for

Correspondence: R.A. Nagourney, Experimental Therapeutics, Memorial Cancer Institute, 2801 Atlantic Avenue, Long Beach, CA 90806, USA.
Received 3 June 1992; and in revised form 18 August 1992.

4 days at 37°C in an atmosphere containing 5% $CO_2$. Following the incubation, 100 µl nigrosin/fast green dye containing 37,500 acetaldehyde-fixed red blood cells (DRBCs) was added to each culture tube, which was briefly vortexed. After 10 min, samples were cytocentrifuged onto glass slides, air-dried and stained with a Wright/Giemsa stain. Cell survival was determined as the ratio of living tumour cells over simultaneously counted DRBCs for each slide using a Whipple disc, with cell survival of drug-treated samples being expressed as a percentage of the saline control values. Our experience has shown that multiple myeloma specimens maintain higher viability in RPMI 1640 enriched with 40% (v/v) horse serum. For the purpose of this study both 15%-FCS and 40%-horse-sera containing media were used in different myeloma samples.

*Statistical analysis*

Statistical analyses were performed using BMDP Statistical Software (BMDP Statistical Software, 1440 Sepulveda Blvd., LA, CA. 90025). Analysis of variance (ANOVA) was used to determine overall differences between samples grouped by disease. The non-parametric (Kruskall-Wallace) test was also applied for comparison with the ANOVA results. The Duncan Multiple Range Test (confidence level set at 95%) and Tukey Studentized Range Method were used to further identify differences between specific disease groups. Correlation coefficients were used to compare the activity of 2-CDA with other classes of chemotherapeutics.

### Results

A comparison of the mean and median $IC_{50}$ values and ranges for 2-CDA in each of the disease types tested is provided in Figure 1. All $IC_{50}$s were calculated from 5 or 10 point dose response curves, using the median effect method of Chou and Talaly (1987). The relatively similar $IC_{50}$ values for ALL, AML, CLL, HCL and NHL (median range 11.98–22.65 nM; mean range 19.90–86.87 nM) are in sharp contrast to the $IC_{50}$ of multiple myeloma (median 863.64 nM; mean 1750.11 nM). An analysis of variance (ANOVA) for 2-CDA $IC_{50}$ found a significant different between groups (diseases), $F(4,41) = 4.64$ ($P < 0.01$). Further analysis using a Duncan Multiple Range Test (95% confidence level) identified multiple myeloma as significantly different from all other disease groups. The Tukey Studentized Range Method indicated that multiple myeloma was significantly different from NHL and CLL ($P < 0.01$); and from ALL and AML ($P < 0.05$). An ANOVA for the $IC_{70}$ of 2-CDA also found a significant difference ($P < 0.001$) between groups, $F(4,41) = 8.96$. Applying the Duncan Multiple Range Test (95% confidence level), multiple myeloma was again identified as being significantly different from all other disease groups. To confirm the ANOVA findings we also applied a non-parametric test (Kruskal-Wallis) which indicated that the $IC_{50}$ value for Multiple myeloma was significantly different from all other groups ($P = 0.0026$).

Pearson sample correlation coefficients (*r*-values) were calculated for each two drug comparison i.e. ARA C *vs* 2-CDA, doxorubicin *vs* 2-CDA, nitrogen mustard *vs* 2-CDA, and vincristine *vs* 2-CDA and are provided in Figure 2. Significance levels for each comparison were determined by Two-Tailed T. When all tumour types are included, the correlation coefficient for nitrogen mustard *vs* 2-CDA is 0.82, for an N of 33 ($P < 0.001$) while all other drugs do not indicate significant correlation with 2-CDA. A comparison of the *r*-values for the highly 2-CDA resistant multiple myeloma samples and relatively 2-CDA sensitive CLL samples is provided at the bottom of Figure 2. Despite wide variation in the 2-CDA $IC_{50}$s for these two disease types, the correlation with nitrogen mustard persists.

To assess the impact of protein binding upon 2-CDA activity in multiple myeloma specimens, we correlated the *in vitro* serum concentrations with the 2-CDA $IC_{50}$ values for eight multiple myeloma specimens. Samples were studied in 15% (v/v) Fetal Calf Serum or 40% (v/v) Horse Serum. Wilcoxon rank sum results indicated a $P$ value = 0.39, which does not support a relationship between serum concentration and $IC_{50}$ for 2-CDA.

**Figure 1** Comparison of 2-CDA $IC_{50}$ values in human hematologic neoplasms: Mean and median $IC_{50}$ values are provided for each tumour type: Acute Lymphoblastic Leukaemia (ALL); Acute Myelogenous Leukaemia (AML); Non Hodgkin's Lymphoma (NHL); Hairy Cell Leukaemia (HCL); Chronic Lymphocytic Leukaemia (CLL); Multiple Myeloma (MYE). Sample $IC_{50}$ Ranges (nM): ALL = 0.12–115.8; AML = 1.34–50.0; CLL = 0.01–250; NHL = 0.01–422; HCL = 17.2–50.0; MYE = 50.0–8,004.

**Figure 2** Correlation coefficients of each 2-drug comparison: Tumour specimens in which multiple drugs were studied in parallel provide cytotoxicity result correlations. Cytarabine (ARA-C); Doxorubicin (DOX); Nitrogen Mustard (NM); Vincristine (VCR). Lower portion of Figure 2 shows correlations between 2-CDA and NM in Chronic Lymphocytic Leukaemia (CLL) and Multiple Myeloma (MYE).

### Discussion

Preliminary observations *in vitro* with 2-CDA conducted between 1984 and 1985 at the Scripps Clinic and Research Foundation and reported in abstract (Nagourney *et al.*, 1989) indicated significant cytotoxicity in a variety of human hematologic neoplasms. This was consistent with the clinical activity originally described in Phase I by Carson *et al.* (1984) and confirmed in Phase II trials (Piro *et al.*, 1988; Piro *et al.*, 1990; Santana *et al.*, 1991; Santana *et al.*, 1992; Kay *et al.*, 1992). Despite 2-CDA's activity in several lymphatic neoplasms, initial *in vitro* studies revealed little or no activity in the B cell neoplasm multiple myeloma (Nagourney *et al.*, 1989). The current report describes observations from 50 specimens of human hematologic tumours and confirms the lack of *in vitro* activity for 2-CDA in multiple myeloma. The relatively similar $IC_{50}$ ranges for both myeloid and most lymphoid neoplasms are in sharp contrast to the mean $IC_{50}$ of multiple myeloma specimens which is two orders of magnitude greater ($P<0.01$). Although non specific protein binding was considered, experiments conducted in which the *in vitro* serum concentrations ranged from 15% FCS (v/v) to 40% Horse serum (v/v) revealed no measurable impact upon cytotoxicity.

The *in vitro* activity of 2-CDA in both myeloid and lymphoid neoplasms indicated by relatively similar $IC_{50}$ ranges suggests potentially broad clinical activity. Avery *et al.* (1989) described significant activity for 2-CDA in cell lines derived from T, B, and non-T/non-B lineages. The significant degree of variance between cell lines was similar to the inter-specimen variability evident in our primary cultures studies. 2-CDA has demonstrated clinical activity in Chronic Lymphocytic Leukemia (Piro *et al.*, 1988) Hairy Cell Leukemia (Piro *et al.*, 1990), Acute Leukemia (Santana *et al.*, 1991; Santana *et al.*, 1992) and low grade lymphomas (Kay *et al.*, 1992). The current report is consistent with these observations. The significant difference observed *in vitro* between multiple myeloma and other tumour types however, strongly suggests that the clinical activity spectrum does not extend to this neoplasm. We recently reported that the multiple myeloma is significantly more resistant *in vitro* to ARA C than other hematologic neoplasms as well (Nagourney *et al.*, 1991). Both 2-CDA and ARA C are substrates for 2-deoxycytidine kinase which could theoretically underlie the observed correlation in this disease. Inadequate sample size of myeloma specimens tested in parallel against both ARA C and 2-CDA in the current study precluded calculation of a myeloma-specific correlation for these two drugs.

We compared the *in vitro* activity of 2-CDA with that of ARA C, vincristine, nitrogen mustard and doxorubicin to assess cross resistance patterns. Pemble *et al.* (1987) reported non-cross resistance between ARA C and 2-bromodeoxyadenosine (2-BDA) in human myeloid leukaemia. Similarly, we found no correlation between 2-CDA and ARA C nor between 2-CDA and vincristine or doxorubicin. However, correlation was observed between 2-CDA and the alkylating agent nitrogen mustard. Subset analysis performed upon the relatively 2-CDA-sensitive CLL specimens and highly 2-CDA-resistant multiple myeloma specimens indicated that cross resistance was present in both data sets despite their widely different $IC_{50}$'s for 2-CDA. Avery (1989) identified synergy between 2-BDA and AZQ in cell lines *in vitro*. Of interest, AZQ is believed to act by alkylation (Akhatar *et al.*, 1975). In preliminary experiments, we have now identified true synergy, by isobologram and computer analysis (Chou & Talalay, 1987), between 2-CDA and the alkylator nitrogen mustard in 4/4 specimens of CLL. Of interest, this degree of synergy was not observed between 2-CDA and gamma irradiation in which only 1/4 CLL specimens revealed synergy and only at suprapharmacologic concentrations. These observations suggest a relationship between the modes of action or mechanisms of resistance of 2-CDA and the mustard alkylators which may not extend fully to gamma irradiation, i.e. depurination.

Conceptually, a laboratory assay which assesses the activity of a chemotherapy drug by measuring total cell kill in a largely non-dividing population of cells should be valid and correlate with clinical outcome if (i) the mechanisms of cell kill *in vitro* are related and proportional to the mechanisms of cell kill *in vivo* or (ii) the mechanisms of cellular resistance *in vitro* are related and proportional to the mechanisms of resistance *in vivo*.

Prior investigations have shown that 2-CDA causes significant cell kill in non dividing populations (Seto *et al.*, 1985; Carson *et al.*, 1983). Seto *et al.* reported that exposure to 2-CDA results in activation of poly ADP ribose polymerase with resultant depletion of cellular NAD and subsequent cell death (Seto *et al.*, 1985). Activation of Poly ADP ribose polymerase has been identified following radiation and alkylator exposure (Berger *et al.*, 1979; Smulson *et al.*, 1977; Berger, 1985). 2-CDA has also been shown to inhibit DNA polymerases, induce depurination and to result in double-strand DNA breaks (Hentosh *et al.*, 1990; Griffig *et al.*, 1989; Tanabe *et al.*, 1989). These activities are all associated with the actions of alkylators. The chemical structure of 2-CDA does not suggest a free-radical mechanism as its mode of action nor as a basis for cross resistance with alkylators. However, activation of Poly ADP ribose polymerase, inhibition of DNA polymerases, single or double strand breaks, depurination or as-yet-unrecognised actions could underlie the observed cross resistance pattern. If confirmed, these results suggest novel therapeutic strategies utilising combinations of alkylators and 2-CDA as promising directions for future clinical trials.

In conclusion, this report confirms our original observations utilising the DiSC assay (Nagourney *et al.*, 1989) regarding the *in vitro* activity spectrum of 2-CDA in primary cultures of human neoplasms. The current report describes *in vitro* activity for this agent in a variety of human hematologic neoplasms with the distinct exception of multiple myeloma. Within given tumour types inter-specimen variation was also observed. Finally, we have observed significant cross resistance between 2-CDA and nitrogen mustard and preliminary evidence of synergy between these agents. The DiSC assay has previously been shown to correlate with both response and survival in a variety of human neoplasms. In this regard, it may serve as a conduit for newly derived agents to enter clinical testing predicated upon the activity spectra identified *in vitro*. Since the time of our original *in vitro* studies with 2-CDA, several clinical trials have established the activity for this drug in many of the neoplasms that we had examined and reported upon in abstract form. At the time of this writing we are unaware of any published data regarding the clinical activity of 2-CDA in multiple myeloma. We await the completion of further clinical trials to allow a prospective assessment of the predictive accuracy of these *in vitro* observations.

The authors would like to thank Drs Jonathan Blitzer, Anthony Ciarolla, Leroy Fass, Herman Kattlove, John Link and William Lyons and the staff of Oncology-Hematology Consultants for providing tumour samples. We are grateful to Dr Wendy Dorchester for her superb assistance with statistical considerations. We thank Dr Gale Granger and Robert Yamamoto for their support and helpful discussions and Darcie Olk for assistance with manuscript preparation. Supported by The Memorial Medical Center Foundation of Long Beach.

### References

AKHTAR, M.F., BAGLEITER, A. & JOHNSON, B. (1975). Studies related to anti-tumor antibiotics. Part IV: Correlation of covalent cross-linking of DNA by bifunctional aziridinoquinones with their antineoplastic activities. *Can. J. Chem.*, **53**, 2891–2897.

AVERY, T.L., REHG, J.E., LUMM, W.C., HARWOOD, F.C., SANTANA, V.M. & BLAKLEY, R.L. (1989). Biochemical pharmacology of 2-chlorodeoxyadenosine in malignant human hematopoietic cell lines and therapeutic effects of 2-bromodeoxyadenosine in drug combinations in mice. *Cancer Res.*, **49**, 4972–4978.

BERGER, N. (1985). Poly (ADP-Ribose) in the cellular response to DNA damage. *Radiat. Res.*, **101**, 4–15.

BERGER, N.A., SIKORSKI, G.W., PETZOLD, S.J. & KUROHARA, K.K. (1979). Association of poly (adenosine diphosphoribose) synthesis with DNA damage and repair in normal human lymphocytes. *J. Clin. Invest.*, **63**, 1164–1168.

BIRD, M.C., BOSANQUET, A.G., FORSKITT, S. & GILBY, E.D. (1986). Semi-micro adaptation of a 4-day differential staining cytotoxicity (DiSC) assay for determining *in vitro* chemosensitivity of haematological malignancies. *Leuk. Res.*, **10**, 445–449.

BOSANQUET, A.G. (1985). Stability of solutions of antineoplastic agents during preparation and storage for in vitro assays. General considerations, the nitrosoureas and alkylating agents. *Cancer Chemother. Pharmacol.*, **14**, 83–95.

BOSANQUET, A.G. (1991). Correlations between therapeutic response of leukaemias and *in vitro* drug-sensitivity assay. *The Lancet*, **337**, 711–714.

CARRERA, C.J., TERAI, C., LOTZ, M., CURD, J.G., PIRO, L.D., BEUTLER, E. & CARSON, D.A. (1990). Potent toxicity of 2-chlorodeoxyadenosine toward human monocytes *in vitro* and *in vivo*. A novel approach to immunosuppressive therapy. *J. Clin. Invest.*, **86**, 1480–1488.

CARRERA, C.J., YAMANAKA, H., PIRO, L.D., LOTZ, M. & CARSON, D.A. (1989). Profound toxicity of deoxyadenosine and 2-chlorodeoxyadenosine toward human monocytes *in vitro* and *in vivo*. *Adv. Exp. Med. & Biol.*, **253b**, 219–225.

CARSON, D.A., WASSON, D.B. & BEUTLER, E. (1984). Antileukemic and immunosuppressive activity of 2-chloro-2′-deoxyadenosine. *Proc. Natl Acad. Sci.*, **81**, 2232–2236.

CARSON, D.A., WASSON, D.B., KAYE, J., ULLMAN, B., MARTIN, Jr, D.W., ROBINS, R.K. & MONTGOMERY, J.A. (1980). Deoxycytidine kinase-mediated toxicity of deoxyadenosine analogs toward malignant human lymphoblasts in vitro and toward murine L1210 leukemia *in vivo*. *Proc. Natl Acad. Sci.*, **77**, 6865–6869.

CARSON, D.A., WASSON, D.B., TAETLE, R. & YU, A. (1983). Specific toxicity of 2-chlorodeoxyadenosine toward resting and proliferating human lymphocytes. *Blood*, **62**, 737–743.

CHOU, T.C. & TALALAY, P. (1987). Applications of the median-effect principle for the assessment of low-dose risk carcinogens and for the quantitation of synergism and antagonism of chemotherapeutic agents. In *New Adventures in Developmental Cancer Chemotherapy*-Bristol-Myers Symposium Series. Harrap, K.R. & Connors, T.A. (eds). pp 37–64, Academic Press.

GAZDAR, A.F., STEINBERG, S.M., RUSSELL, E.K., LINNOILA, R.I., OIE, H.K., GHOSH, B.C., COTELINGAM, J.D., JOHNSON, B.E., MINNA, J.D. & IHDE, D.C. (1990). Correlations of *in vitro* drug-sensitivity testing results with response to chemotherapy and survival in extensive stage small cell lung cancer. A prospective clinical trial. *J. Natl Cancer Inst.*, **82**, 117–123.

GRIFFIG, J., KOOB, R. & BLAKLEY, R.L. (1989). Mechanisms of inhibition of DNA synthesis by 2-chlorodeoxyadenosine in human lymphoblastic cells. *Cancer Res.*, **49**, 6923–6928.

HENTOSH, P., RAINER, K. & BLAKLEY, R.L. (1990). Incorporation of 2-Halogeno-2′-deoxyadenosine 5-Triphosphates into DNA during replication by human polymerases α and β. *J. Biol. Chem.*, **265**, 4033–4040.

KAY, S.C., SAVEN, A., CARRERA, C.J., CARSON, D.A., THURSTON, D., BEUTLER, E. & PIRO, L.D. (1992). 2-Chlorodeoxyadenosine treatment of low-grade lymphomas. *J. Clin. Oncol.*, **10**, 371–377.

LATHAN, B., VON-TETTAU, M., VERPOORT, K. & DIEHL, V. (1990). Pretherapeutic drug testing in acute leukemias for prediction of individual prognosis. *Hamatol. Bluttransfus.*, **33**, 295–298.

NAGOURNEY, R.A., LINK, J., SCHLUTZ, M. & WEISENTHAL, L.M. (1991). Cytarabine (ARA-C) activity in chronic lymphocytic leukemia (CLL). Laboratory observations with potential clinical applications. *Proc. Amer. Soc. Clin. Oncol.*, **10**, 229.

NAGOURNEY, R.A., MESSENGER, J.C. & EVANS, S.S. (1989). 2-Chlorodeoxyadenosine (2-CDA) activity in primary cultures of human hematologic neoplasms (HN). *Proc. Am. Assoc. Cancer Res.*, **30** abs 2434.

PEMBLE, L.B., LIHOU, M.G., BLAKLEY, R.L., JAMIESON, G.P. & SMITH, P.J. (1987). Lack of cross resistance between cytosine arabinoside and a new halogenated nucleoside analogue, 2-bromo-2′-deoxyadenosine in human acute myeloid leukemia cells. *Cancer Chemother. Pharmacol.*, **20**, 155–161.

PIRO, L.D., CARRERA, C.J., BEUTLER, E. & CARSON, D.A. (1988). 2-Chlorodeoxyadenosine: an effective new agent for the treatment of chronic lymphocytic leukemia. *Blood*, **72**, 1069–1073.

PIRO, L.D., CARRERA, C.J., CARSON, D.A. & BEUTLER, E. (1990). Lasting remissions in hairy-cell leukemia induced by a single infusion of 2-Chlorodeoxyadenosine. *New England J. Med.*, **322**, 1117–1121.

SANTANA, V.M., MIRRO, Jr. J., HARWOOD, F.C., CHERRIE, J., SCHELL, M., KALWINSKY, D. & BLAKLEY, R.L. (1991). A Phase I clinical trial of 2-Chlorodeoxyadenosine in pediatric patients with acute leukemias. *J. Clin. Oncol.*, **9**, 416–422.

SANTANA, V.M., MIRRO, J., KEARNS, C., SCHELL, M.J., CROM, W. & BLAKLEY, R.L. (1992). 2-Chlorodeoxyadenosine produces a high rate of complete hematologic remission in relapsed acute myeloid leukemia. *J. Clin. Oncol.*, **10**, 364–370.

SETO, S., CARRERA, C.J., KUBOTA, M., WASSON, D.B. & CARSON, D.A. (1985). Mechanism of deoxyadenosine and 2-Chlorodeoxyadenosine toxicity to nondividing human lymphocytes. *J. Clin. Invest.*, **75**, 377–383.

SMULSON, M.E., SCHEIN, P., MULLINS, D.W. Jr & SUDHAKAR, S. (1977). A putative role for nicotinamide adenine dinucleotide-promoted nuclear protein modification in the antitumor activity of N-methyl-N-nitrosourea. *Cancer Res.*, **37**, 3006–3012.

TANABE, K., HIRAOKA, W., KUWABARA, M., SATO, F., MATSUDA, A. & UEDA, T. (1989). Induction of DNA double-strand breaks in Chinese hamster V19 cells by 2-Chlorodeoxyadenosine. *Chem.-Biol. Interactions*, **71**, 167–175.

TIDEFELT, U., SUNDMAN-ENGBERG, B., RHEDIN, A.S. & PAUL, C. (1989). *In vitro* drug testing in patients with acute leukaemia with incubations mimicking *in vivo* intracellular drug concentrations. *European J. Hematol.*, **43**, 374–384.

WEISENTHAL, L.M., MARSDEN, J.A., DILL, P.L. & MACALUSO, C.K. (1983). A novel dye exclusion method for testing in vitro chemosensitivity of human tumors. *Cancer Res.*, **43**, 749–753.